# Global gene expression profiling displays a network of dysregulated genes in non-atherosclerotic arterial tissue from patients with type 2 diabetes

**DOI:** 10.1186/1475-2840-11-15

**Published:** 2012-02-17

**Authors:** Vibe Skov, Steen Knudsen, Malene Olesen, Maria L Hansen, Lars M Rasmussen

**Affiliations:** 1 Department of Clinical Biochemistry and Pharmacology, Odense University Hospital, Odense, Denmark; 2 Department of Clinical Genetics, Odense University Hospital, Odense, Denmark; 3 Medical Prognosis Institute A/S, Hørsholm, Denmark; 4 Department of Cardiothoracic and Vascular Surgery, Odense University Hospital, Denmark

**Keywords:** Systems biology, Microarray, Diabetes mellitus, Gene expression, Coronary artery disease

## Abstract

**Background:**

Generalized arterial alterations, such as endothelial dysfunction, medial matrix accumulations, and calcifications are associated with type 2 diabetes (T2D). These changes may render the vessel wall more susceptible to injury; however, the molecular characteristics of such diffuse pre-atherosclerotic changes in diabetes are only superficially known.

**Methods:**

To identify the molecular alterations of the generalized arterial disease in T2D, DNA microarrays were applied to examine gene expression changes in normal-appearing, non-atherosclerotic arterial tissue from 10 diabetic and 11 age-matched non-diabetic men scheduled for a coronary by-pass operation. Gene expression changes were integrated with GO-Elite, GSEA, and Cytoscape to identify significant biological pathways and networks.

**Results:**

Global pathway analysis revealed differential expression of gene-sets representing matrix metabolism, triglyceride synthesis, inflammation, insulin signaling, and apoptosis. The network analysis showed a significant cluster of dysregulated genes coding for both intra- and extra-cellular proteins associated with vascular cell functions together with genes related to insulin signaling and matrix remodeling.

**Conclusions:**

Our results identify pathways and networks involved in the diffuse vasculopathy present in non-atherosclerotic arterial tissue in patients with T2D and confirmed previously observed mRNA-alterations. These abnormalities may play a role for the arterial response to injury and putatively for the accelerated atherogenesis among patients with diabetes.

## Background

Cardiovascular diseases (CVD) in patients with type 2 diabetes are a large and increasing health problem. Increased atherosclerotic lesions are believed to form the basis behind the high frequency of CVD in diabetes; however, epidemiological studies have shown that traditional risk factors, e.g. hypertension and dyslipidemia cannot explain the increased incidence [1]. Unfortunately, the molecular mechanisms leading to atherosclerosis in diabetes are only partially understood [2].

The arterial wall in diabetes harbors not only increased amounts of atherosclerotic plaques, but also diffuse alterations present in non-atherosclerotic parts of the vessel wall. One element of the generalized alterations in the vasculature in diabetes is endothelial dysfunction [3], characterized by increased permeability [4], increased expression of pro-inflammatory molecules [5], and altered vasomotoric responses [6]. Moreover, changes in extracellular matrix components of the tunica media are present in both atherosclerotic and non-atherosclerotic parts of the arterial tree in diabetes. Increased concentrations of collagen type 4 [7], hyaluronic acid [8], osteopontin, osteoprotegerin [9], and metalloproteinases [10] have, for example, been described in conjunction with the presence of high amounts of glucose-derived increased cross-linking of collagens [11]. Decreased amounts of several gene products related to apoptosis have been observed in vascular smooth muscle cells from normal appearing areas of arteries from patients with diabetes [12]. In addition, linear media calcifications occur with increased frequency among patients with glucose intolerance and diabetes and are strong predictors of CVD in these individuals [13,14]. In accordance, recent studies of non-atherosclerotic arterial alterations in animal models of type 2 diabetes and hyperglycemia demonstrated increased aortic stiffness and upregulation of matrix components [15], increased arterial calcification [16], and accumulation of glycosaminoglycan-rich material [17]. Thus, defects in several molecular pathways seem to be present in the arterial wall in patients with type 2 diabetes. These changes are likely to play important roles in the arterial response to injury and thus in the build-up of atherosclerotic plaques in diabetic patients.

By using DNA microarrays for transcriptional profiling, a large number of genes can be analyzed simultaneously and the resulting data can be integrated with pathways and biological interaction networks to detect coordinated changes in functionally related genes. Recently, we applied gene expression microarrays and identified fibulin-1 (FBLN1) as a molecular marker of cardiovascular disease in non-atherosclerotic tissue from patients with type 2 diabetes [18]. To our knowledge, only one other study has examined global gene expression changes in arterial tissue from diabetic patients; however, the results only reflected different degrees of atherosclerosis and not specific diabetes related alterations [19].

In the present study, we hypothesized that diffuse diabetes-related alterations in non-atherosclerotic arterial tissue are associated with a cluster of dysregulated genes involved in vascular and metabolic cell functions. We applied global pathway and network analysis to unravel the gene expression profile of well-defined, normal-appearing arterial tissue from patients with type 2 diabetes.

## Methods

### Subjects

Study subjects were recruited from patients undergoing artery by-pass graft surgery at Skejby and Odense University Hospital. Participants included ten men with more than 2 years known duration of type 2 diabetes, defined as diabetes diagnosed after adolescence, and eleven non-diabetic men matched according to age (Table 1). Participants and tissue handling have been described in a study aimed at identifying single genes differentially expressed in type 2 diabetes [18]. Briefly, a small piece of arteria mammaria interna from all patients was dissected into intima-media and surrounding tissue and frozen in liquid nitrogen within a few minutes. The arteries are spare material from the repair vessel from coronary by-pass operations. The investigation was approved by the local Danish ethical committee in Region Mid and South and informed consent was obtained from each subject.

**Table 1:** Characteristics of diabetic patients and control subjects

	Diabetic patients	Control subjects
N	10	11
Age (years)	65.6 ± 11.9	65.2 ± 8.5
Systolic blood pressure (mmHg)	143.1 ± 35.6	153.9 ± 16.6
Diastolic blood pressure (mmHg)	76.3 ± 11.6	87.9 ± 8.7^†^
Hemoglobin A1c (fract.)	0.074 ± 0.01	0.060 ± 0.004^††^
Total cholesterol (mmol/l)	4.1 ± 0.8	4.7 ± 0.5
High Density Lipoprotein (mmol/l)	1.2 ± 0.2	1.4 ± 0.3

Data represent means ± SD; Students T-test for non-paired data used. ^†^*P *< 0.05 and ^††^*P *< 0.005 for diabetic patients vs. control subjects

### Histology

Tissue for histology was fixed for 24-72 hours in PBS-buffered 4% formaldehyde and subsequently embedded in paraffin. Sections were cut and stained by hematoxylin-eosin, von kossa, and elastin using routine methods [18].

### RNA isolation and microarray hybridization

Total RNA was isolated and hybridized to microarrays as previously described [18]. Briefly, total RNA was isolated from intima-media samples by using the TRIzol protocol (Life Technologies, Gaithersburg, MD, USA), and purified RNA was converted to biotin-labeled amplified RNA (aRNA) and hybridized to Affymetrix HG-U133A 2.0 chips (Affymetrix, Santa Clara, CA, USA).

### Data processing and statistical analysis

All microarray chips were preprocessed as reported previously [18]. The regularized t-test limma http://www.bioconductor.org was applied to evaluate differences in gene expression between patients with diabetes and control subjects. Genes with an uncorrected *P *< 0.05 were considered for further analysis.

### Global pathway and network analysis

Changes in gene expression at the pathway level between diabetic patients and control subjects were assessed using GO-Elite 1.19 http://www.genmapp.org/go_elite/ and Gene Set Enrichment Analysis (GSEA) 2.0.1 [20]. GO-Elite is a software tool that runs an updated version of MAPPFinder [21] and filters redundant pathways. A total of 113 pathways were analyzed using GO-Elite. A z-score was calculated for each pathway, and 2000 permutations were applied to assess the significance of the z-score http://www.genmapp.org. Applying the MSigDB database http://www.broad.mit.edu/gsea/msigdb in GSEA, we compiled an "artery gene set" consisting of 820 gene sets and pathways. All genes were ranked according to the ttest and an enrichment score (ES) was assigned to each gene set. The statistical significance of the ES was estimated by performing 2000 gene permutations. After gene set enrichment analysis in GSEA, we masked the redundant gene sets by using the leading edge analysis option http://www.broad.mit.edu/gsea.

Gene expression changes between diabetic patients and control subjects were integrated with molecular interaction networks using Cytoscape 2.6.0 [22]. The Human Interactome data set http://www.cytoscape.org/cgi-bin/moin.cgi/Data_Sets, composed of 61,263 protein-protein interactions analogous to 10,000 different proteins, was loaded into the Cytoscape software. To reduce the complexity of the large network, differentially expressed genes from the data set (*P *< 0.05) were mapped onto the network, and then the filter option in Cytoscape was used to select proteins (nodes) interacting directly with the differentially expressed genes generating a new network. To identify hubs--i.e. interacting nodes with high network degrees assumed to correspond to essential gene products in the cell [23]--with 15 or more interactions, the topology filter option was applied to the new network creating a smaller network. The jActiveModules plugin in Cytoscape was applied to the small network to identify a subnetwork with an overall significant response to the experimental condition. The anneal algorithm and 250.000 iterations were used in the jActiveModules plugin to identify the subnetwork. A z-score was calculated for the subnetwork and the significance of the z-score was assessed by performing 1000 permutations. NetworkAnalyzer is a core plugin in Cytoscape and has been used to calculate topological values for each gene in the subnetwork.

## Results

The mean age of the study subjects was 65 years (Table 1). Patients with type 2 diabetes had elevated hemoglobin A1c (*P *< 0.005) and reduced diastolic blood pressure (*P *< 0.05). There was no difference between the two groups with respect to systolic blood pressure, HDL, and total cholesterol as previously reported [18].

### Histology

Tissue from all subjects appeared normal, i.e. without atherosclerosis, calcified areas, or cellular infiltrations, as revealed by von kossa, hematoxylin-eosin, and elastin staining as previously reported [18]. In a few instances, enough tissue was obtained to make additional histology after dissection to control the separation of intima-media from adventitia. In these cases, dissection was made just outside the lamina elastica externa.

### Global gene expression analysis

Of the 22,277 probe sets represented on the array, 477 were downregulated and 169 were upregulated in type 2 diabetic patients versus control subjects as reported previously (*P *< 0.05) [18]. No probe sets were significantly differentially expressed after controlling for multiple hypothesis testing using the Benjamini-Hochberg method [24].

### Analysis of gene sets and biological pathways

To examine the difference in gene expression between the two groups at the pathway level, data were loaded into GSEA 2.0.1 and GO-Elite 1.19. Applying GSEA, caries pulp up (genes upregulated in pulpal tissue from carious teeth) [25], neuroactive ligand receptor interaction, cell adhesion molecules, monocyte dend DN (genes downregulated in monocyte dendritic cells), and cytokine-cytokine receptor interaction were the most significantly upregulated gene sets (FWER < 0.05). The most significantly downregulated gene sets included kidney transplant well UP [26], insulin 2F UP (genes 2 fold upregulated in muscle by insulin) [27], heartfailure atria DN, GH GHRHR KO 24 hrs DN [28], proteasome, and diab neph DN (genes downregulated in the glomeruli of cadaver kidneys from patients with diabetic nephropathy) (FWER < 0.05) (Table 2). Further description of the gene sets can be found in the MsigDB database http://www.broad.mit.edu/gsea/msigdb.

**Table 2:** Ranking of the top 10 up- and downregulated gene sets using GSEA in intima-media of type 2 diabetic patients vs. control subjects

GENE SET	SIZE	ES	NES	NOM	FDR	FWER
				*p*-value	*p*-value	*p*-value
*Upregulated gene sets*						
Caries pulp UP	202	-0.43	-2.29	< 0.0001	0.0006	0.0005
HSA04080 Neuroactive ligand receptor interaction	227	-0.42	-2.27	< 0.0001	0.0009	0.002
HSA04514 Cell adhesion molecules	114	-0.43	-2.18	< 0.0001	0.002	0.005
Monocyte dend DN	115	-0.43	-2.13	< 0.0001	0.003	0.01
HSA04060 Cytokine-cytokine receptor interaction	225	-0.39	-2.12	< 0.0001	0.003	0.01
Kidney transplant rejection UP	80	-0.45	-2.09	< 0.0001	0.004	0.02
HSA04610 Complement and coagulation cascades	63	-0.45	-2.05	< 0.0001	0.007	0.04
HSA01430 Cell communication	108	-0.41	-2.02	< 0.0001	0.008	0.05
HSA04640 Hematopoietic cell lineage	83	-0.43	-2.01	< 0.0001	0.008	0.06
Osteoclasts sig	37	-0.50	-1.94	< 0.0001	0.02	0.13
*Downregulated gene sets*						
Kidney transplant well UP	251	0.56	2.45	< 0.0001	< 0.0001	< 0.0001
Insulin 2F UP	167	0.57	2.41	< 0.0001	< 0.0001	< 0.0001
Heartfailure atria DN	109	0.55	2.20	< 0.0001	0.0002	0.0005
GH GHRHR KO 24 hrs DN	140	0.53	2.18	< 0.0001	0.0001	0.0005
HSA03050 Proteasome	21	0.73	2.16	< 0.0001	0.0002	0.001
Diab neph DN	249	0.49	2.15	< 0.0001	0.0002	0.001
HSA00190 Oxidative phosphorylation	99	0.55	2.15	< 0.0001	0.0001	0.001
Kidney transplant well PBL DN	40	0.61	2.09	< 0.0001	0.0005	0.004
Krebs TCA cycle	29	0.65	2.07	< 0.0001	0.0008	0.008
mRNA processing	41	0.61	2.07	0.0007	0.0008	0.009

Ranking of the gene sets was done using GSEA 2.0.1. ES, enrichment score; NES, normalized enrichment score; NOM, nominal; FDR, false discovery rate; FWER, family wise error rate

The statistical rating of the results in GO-Elite was provided by the z-score, and pathways with a z-score above 2.0 were considered significant [21]. Evaluation of the results revealed significant upregulation of triacylglyceride synthesis, apoptosis, matrix metalloproteinases, and adipogenesis (z > 2.0). Five pathways including cholesterol biosynthesis, TNF-alpha NFkB Netpath 9, Krebs TCA Cycle, non-homologous end joining, and signaling of hepatocyte growth factor receptor were significantly downregulated (z > 2.0) (Table 3).

**Table 3:** Ranking of up- and downregulated pathways in intima-media of type 2 diabetic patients vs. control subjects using GO-Elite 1.19

MAPP Name	Changed	Measured	On MAPP	Changed	Z	Permute	FWER
	(n)	(n)	(n)	(%)	score	*p*-value	*p*-value
*Upregulated pathways*							
Triacylglyceride synthesis	3	22	24	13.64	4.7	0.002	0.23
Apoptosis	4	83	85	4.82	2.5	0.04	0.67
Matrix metalloproteinases	2	30	32	6.67	2.4	0.04	0.67
Adipogenesis	5	129	133	3.88	2.3	0.04	0.67
*Downregulated pathways*							
Cholesterol biosynthesis	4	15	15	26.67	4.8	0.004	0.15
TNFalpha NFkB netpath 9	16	187	208	8.56	3.8	0.003	0.15
Krebs TCA cycle	5	32	39	15.63	3.7	0.007	0.18
Non-homologous end joining	2	7	8	28.57	3.6	0.04	0.61
Signaling of hepatocyte growth factor receptor	5	35	36	14.29	3.5	0.02	0.39

Shown are significantly up- and downregulated pathways in intima-media of diabetic patients vs. control subjects. Only non-redundant pathways with a z-score above 2.0 are listed. A foldchange of 1.05 and a *p*-value < 0.05 were used as filtering criteria. Changed (n): number of changed genes, Measured (n): number of genes on the chip, On MAPP (n): number of genes on the MAPP, Changed (%): percentage of changed genes, FWER: family wise error rate

### Computational analysis of the Human Interactome network

**Figure 1:**
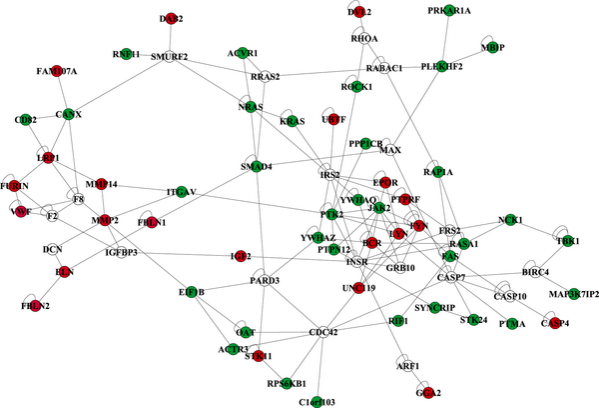
**Interaction subnetwork of potential arterial disease-associated genes in patients with type 2 diabetes**. The subnetwork consists of 74 nodes and 166 edges. Red nodes represent upregulated genes, green nodes represent downregulated genes (*P *< 0.05), and white nodes represent genes with *P *> 0.05. The z-score of the network is 9.23 (permute *P *= 0.01)

To further explore the global gene expression changes associated with arterial disease in type 2 diabetes, data were integrated with the Human interactome network to identify the principal subnetworks i.e. connected regions of the network that show significant changes in expression. 397 differentially expressed genes (*P *< 0.05) from the microarray data set interacted directly with 2980 proteins (nodes) in the network creating a network composed of 3227 interacting nodes. Filtering of hubs with 15 or more interactions resulted in a network consisting of 1043 nodes. Performing jActiveModules on the 1043 node network unveiled 5 highly overlapping subnetworks, therefore, we decided only to focus on the top network consisting of 74 nodes (z = 9.23) (permute *P *= 0.01). The statistically significant 74 node subnetwork is depicted in Figure 1. Topological values and number of interactors for all genes are shown in Table 4. The topological coefficient is a relative measure for the extent to which a node shares neighbors with other nodes. The most important cardiometabolic hubs and their interactors are shown in Table 5.

**Table 4:** Topological values and number of interactors for all genes in the network

Gene	TPV	NOI	Gene	TPV	NOI	Gene	TPV	NOI
ACTR3	0.625	2	FURIN	0.52	3	PTK2	0.20	8
ACVR1	0.7	2	FYN	0.27	6	PTMA	0	1
ARF1	0.5	2	GGA2	0	1	PTPN12	0.51	3
BCR	0.26	6	GRB10	0.39	4	PTPRF	0.36	3
BIRC4	0.33	3	IGF2	0.5	2	RABAC1	0.25	4
C1orf103	0	1	IGFBP3	0.33	3	RAP1A	0.33	3
CANX	0.27	5	INSR	0.17	10	RASA1	0.22	5
CASP10	0.33	3	IRS2	0.16	8	RHOA	0.33	3
CASP4	0	1	ITGAV	0.39	3	RIF1	0.5	2
CASP7	0.13	8	JAK2	0.24	8	RNF11	0	1
CD82	0.71	2	KRAS	0.59	2	ROCK1	0.5	2
CDC42	0.16	8	LRP1	0.32	5	RPS6KB1	0.57	2
DAB2	0	1	LYN	0.31	5	RRAS2	0.3	4
DCN	0.5	2	MAP3K7IP2	0	1	SMAD4	0.23	5
DVL2	0	1	MAX	0.25	4	SMURF2	0.24	5
EIF1B	0.31	4	MBIP	0	1	STK11	0.63	2
ELN	0.33	3	MMP14	0.41	3	STK24	0.5	2
EPOR	0.42	3	MMP2	0.32	4	SYNCRIP	0.5	2
F2	0.41	4	NCK1	0.39	3	TBK1	0.5	2
F8	0.31	5	NRAS	0.25	5	UBTF	0	1
FAM107A	0	1	OAT	0.63	2	UNC119	0.33	4
FAS	0.25	4	PARD3	0.24	5	VWF	0.57	3
FBLN1	0.5	2	PLEKHF2	0.25	4	YWHAQ	0.55	2
FBLN2	0	1	PPP1CB	0.5	2	YWHAZ	0.36	3
FRS2	0.39	3	PRKAR1A	0	1			

TPV: topological value; NOI: number of interactors. The TPV is a number between 0-1. Nodes that have one neighbor are assigned a topological value of 0

**Table 5:** Important cardiometabolic hubs and their interactors in non-atherosclerotic diabetic arterial tissue

Hub	Interactor	Hub	Interactor	Hub	Interactor	Hub	Interactor
**INSR**	ARF1	**CDC42**	ACTR3	**IRS2**	EPOR	**SMAD4**	FBLN1
	FRS2		C1ORF103		INSR		MAX
	GRB10		CASP7		KRAS		NRAS
	IGF2		OAT		NRAS		PARD3
	IRS2		PARD3		PTPRF		RRAS2
	JAK2		RIF1		UBTF	**MMP2**	DCN
	PTK2		RPS6KB1		YWHAQ		IGFBP3
	PTPN12		UNC119		YWHAZ		ITGAV
	RASA1						MMP14
	SYNCRIP						

## Discussion

In the present study, we used high-density oligonucleotide arrays to elucidate global gene expression patterns in non-atherosclerotic, non-calcified, normal-appearing arterial tissue from patients with type 2 diabetes. Previously, we used these data in a study aimed at identifying new molecular markers of arterial disease in type 2 diabetes. Examinations were done at the single gene level with microarrays, and we were able to validate several genes with q-RT-PCR including FBLN1 as significantly upregulated in the vascular wall both at the mRNA and protein level [18]. In the present bioinformatic study, we use our mRNA expression data to explore biological pathways and networks that are dysregulated in the arterial wall in diabetes. Although our data indicate that differences in gene expression between arterial tissue from patients with diabetes and non-diabetic subjects are modest at the single gene level [18], we demonstrate that clusters of highly interconnected genes are significantly dysregulated at the transcriptional level in arterial tissue in diabetes. Providing credibility to our results, a number of gene products of these pathways has previously been found dysregulated in the vessel wall in diabetic vasculopathy, as discussed below.

The results from GSEA demonstrate significant upregulation of gene sets and pathways related to cytokine-growth factor- and hormonal actions in arterial tissue from diabetic individuals. This include the gene sets caries pulp up, cell adhesion molecules, monocyte dend DN, and cytokine-cytokine receptor interaction with caries pulp up as the most significantly upregulated gene set. Interestingly, there is an increasing evidence supporting an association between periodontitis and diabetic complications [29,30] suggesting that genes related to periodontitis could play a role for the development of diabetic vasculopathy. Because the tissue in our study is without atherosclerosis and cellular infiltration, it seems that the vascular smooth muscle cells themselves may express an inflammatory phenotype. In agreement, previous observations showed that vascular smooth muscle cells produce a range of peptide factors that have been suggested to play a role in diabetic vasculopathy [9]. In addition, this finding is in line with previous observations that vascular cells in diabetes may display increased inflammatory capacity, at least partly related to effects of toll-like receptors [31,32].

Over the past decades there has been much debate regarding the relative importance of hyperinsulinism and insulin resistance in vascular cells from diabetic patients [33-36]. Interestingly, the insulin 2F-pathway, representing genes upregulated 2-fold by insulin stimulation of skeletal muscle in healthy subjects [27], was significantly downregulated in our study, supporting the idea that at least some effects of insulin in vascular tissue are influenced by insulin resistance and not by hyperinsulinism. This is in agreement with several human and experimental observations [37-39].

Surprisingly, a triglyceride pathway was the most significantly upregulated pathway using GO-Elite. Several organs, such as muscle and liver, are known to accumulate triglycerides in type 2 diabetes [40]; however, no information concerning arterial tissue is currently available. Our data are compatible with the hypothesis that accumulation of triglycerides could also take place in the arterial wall.

Since the apoptotic process has been found to be regulated in vascular smooth muscle cells from arterial tissue from individuals with type 2 diabetes [12], it is of interest that our data demonstrate an increased expression of the apoptotic pathway using GO-Elite. The apoptotic pathway may be regulated in arterial tissue in diabetes; however, further studies are warranted to assess the precise implication of apoptosis in the pathogenesis of diabetic arteriopathy. Of interest, also a pathway defined as genes downregulated in glomeruli from patients with diabetic nephropathy was downregulated in the arterial wall in diabetes [41]. This observation may be an indication of a common set of dysregulated genes present in both micro- and macro-angiopathy in diabetes.

Although many detailed unique processes are suggested to be implicated in the development of arterial disease in diabetes, an understanding of the disease mechanisms as an integrated whole may be warranted. Network analysis provides a broad insight into biology in the context of known functional interrelationships among proteins. Our network analysis demonstrates a statistically significant cluster of genes that are dysregulated in the arterial wall in diabetes.

Looking for important hubs in the network, it is interesting to note that the insulin receptor (INSR) appears, although it is not itself regulated. It seems that many genes, interacting with the receptor, are indeed regulated in the arterial wall in diabetes, which is in accordance with the idea that dysfunctional effects of insulin may play a role in diabetic arterial disease (35-37). Another relevant hub is SMAD4, which seems to have an important place in the network, interacting both with intracellularly expressed genes like RRAS2 and PARD3, as well as an extracellular matrix gene, fibulin-1 (FBLN1). SMAD4 is an important intracellular signaling molecule in the TGF-beta system which is in agreement with other observations showing that this system is involved in the development of matrix accumulations in diabetic complications [42,43]. A recent transcriptome analysis of human diabetic kidney disease pointed towards CDC42 signaling as an important dysregulated pathway [44], which is in accordance with the presence of CDC42 as an important hub in the network we present here. MMP2 is an important hub in the network with connections to other relevant genes including MMP14, DCN and ITGAV. This part of the network is in agreement with other previously reported data showing that MMP2 is dysregulated in the arterial wall in diabetes [45] and seems to indicate that matrix remodeling may be an important feature of the non-atherosclerotic arterial disease seen in diabetes.

## Conclusions

We used transcriptional profiling on well-defined non-atherosclerotic arterial samples from diabetic individuals. Using pathway and network analysis, our data display a statistically significant cluster of dysregulated genes in the arteries of diabetic patients, which is in accordance with the presence of a diffuse diabetic macroangiopathy, similar to the diabetic microangiopathy. Our approach has not previously been used, but point towards dysregulated pathways related to matrix metabolism, triglyceride synthesis, inflammation, as well as insulin signaling and apoptosis. Dysregulated gene interactions and pathways in the cells of the arterial wall in diabetes may play important roles in the arterial response to injury and atherosclerosis.

## Abbreviations

GSEA: Gene Set Enrichment Analysis; ES: Enrichment score; VWF: von Willebrand factor; LEP: Leptin; MMP2: Matrix metallopeptidase 2; FBLN1: Fibulin1; FBLN2: Fibulin2; EGR3: Early growth factor 3; HBA2: Hemoglobin alpha 2; FAS: TNF receptor superfamily, member 6; TNFAIP6: Tumor necrosis factor, alpha-induced protein 6; ELN: Elastin

## Competing interests

The authors declare that they have no competing interests.

## Authors' contributions

VS participated in the design of the study, performed the bioinformatic analysis and drafted the manuscript. SK participated in the design of the study and in interpretation of data. MO and MLH collected and organized patient data. LMR participated in the design of the study and in interpretation of data, and drafted the manuscript. All authors read and approved the final manuscript.

## Data deposition footnote

Data are available from GEO (http://www.ncbi.nlm.nih.gov/geo, Accession No. GSE13760).
